# The Factors Influencing Pregnant Women’s Selection of Media Sources to Obtain Information on COVID-19 in Japan in 2021

**DOI:** 10.3390/vaccines11040805

**Published:** 2023-04-06

**Authors:** Shihoko Komine-Aizawa, Naotake Yamada, Yasuo Haruyama, Masashi Deguchi, Mitsuru Fukuda, Kei Kawana, Gen Kobashi, Etsuko Miyagi, Hideto Yamada, Takashi Sugiyama, Satoshi Hayakawa

**Affiliations:** 1 Division of Microbiology, Department of Pathology and Microbiology, Nihon University School of Medicine, Tokyo 173-8610, Japan; 2 Institute of Journalism and Media, Nihon University, Tokyo 101-8375, Japan; 3 Integrated Research Faculty for Advanced Medical Sciences, Dokkyo Medical University, Tochigi 321-0293, Japan; 4 Department of Obstetrics and Gynecology, Kobe University Graduate School of Medicine, Kobe 650-0017, Japan; 5 College of Risk Management, Nihon University, Tokyo 154-8513, Japan; 6 Division of Obstetrics and Gynecology, Nihon University School of Medicine, Tokyo 173-8610, Japan; 7 Department of Public Health, Dokkyo Medical University, Tochigi 321-0293, Japan; 8 Department of Obstetrics and Gynecology, Yokohama City University Hospital, Yokohama 236-0004, Japan; 9 Center for Recurrent Pregnancy Loss, Teine Keijinkai Hospital, Sapporo 006-8555, Japan; 10 Department of Obstetrics and Gynecology, Ehime University Graduate School of Medicine, Toon 791-0295, Japan

**Keywords:** COVID-19, pregnancy, media, vaccine

## Abstract

Pregnant women presumably gather information about the coronavirus disease 2019 (COVID-19) from various sources. However, it is difficult for pregnant women who are not medical professionals to source the appropriate information because of the infodemic related to the COVID-19 pandemic. Therefore, the objective of our study was to investigate how pregnant women gathered information about COVID-19 and COVID-19 vaccination. To address this issue, we conducted an online questionnaire survey between 5 October and 22 November 2021, which was approved by the Ethics Committee of Nihon University School of Medicine. We received 4962 responses after excluding 1179 insufficient answers. Our study found that age, occupation, and infection-risk anxiety influenced the selection of media for obtaining information. Pregnant women who were older, medical professionals, public servants, or educators tended to rely on specialized medical websites, whereas housewives tended to use mass media, social media, and sources with uncertain scientific evidence. Additionally, the number of weeks of gestation and the method of conception (natural or assisted reproductive conception) affected the selection of media. The accessibility of COVID-19 information for pregnant women was determined by their social background and pregnancy status. We need to continue making efforts to ensure that appropriate information is readily available to pregnant women and their families.

## 1. Introduction

The coronavirus disease 2019 (COVID-19) pandemic has impacted people around the world, including pregnant women. Many pregnant women tend to obtain various information to protect their own and their families’ health. For nonmedical professionals, the media is the primary source of information about health, including infectious diseases and prevention [1]. In general, “media” often means mass media (newspapers, radio, and television). Mass media can be defined as a medium through which a specific type of information or news group disseminates information in one direction to an unspecified number of people. Recently, there has been a remarkable development in social media, such as Facebook, Twitter, Instagram, and YouTube, since social media allows anyone, including those without specialized knowledge, to easily disseminate information. While social media is easily accessible to users, there is a risk that the reliability of information obtained through social media is low.

The COVID-19 pandemic has brought physical health challenges and a surge in information, leading to an “infodemic”. This information overload, coupled with the spread of misinformation by the media, has created confusion and anxiety among pregnant women about COVID-19 and the COVID-19 vaccine. So far, it has been reported that pregnant women have a strong fear of COVID-19 [2,3] and the COVID-19 vaccine [3,4]. In addition, under the COVID-19 burden, stress among pregnant women was reported to increase [5,6,7]. It has also been reported that pregnant women’s fear and anxiety about COVID-19 correlate with their use of websites and social media. [8]. According to the scoping review by De Brabandere et al., maternal vaccination heavily relies on the internet and social media as important sources of information [9]. Meanwhile, medical and administrative professionals, including gynaecologists and obstetricians, have continued their efforts to disseminate information to the general public, who are not medical professionals, via society websites and other means in order to communicate accurate information to pregnant women. It is unknown, however, which media pregnant women use to gather information and which media they deem effective. Personal backgrounds play a role in individuals’ selection of media, including pregnant women who consider various factors such as their sociodemographic characteristics (e.g., age, occupation), pregnancy stage, previous birth history, type of pregnancy (single or multiple fetuses, natural or assisted), and concerns about infectious diseases and vaccine safety. We hypothesized that pregnant women with multiple fetuses or medical complications would seek additional information. In the same manner, pregnant women who have significant concerns about infection with COVID-19 or immunizations may seek alternative information sources than other pregnant women. Although it has been reported that “media” can influence the anxiety of pregnant women about COVID-19 and their vaccination behaviour [9], it is unclear how pregnant women with specific backgrounds select which media to obtain information about COVID-19 and vaccination. Although it has already been reported that COVID-19 vaccination during pregnancy can suppress severe symptoms in pregnant women who have been infected with SARS-CoV-2 [10,11], there are a certain number of pregnant women who have not received the vaccine due to vaccine hesitancy. It has been noted that the information flowing from various media publications and social media affects people’s behavior regarding vaccination, including vaccine reluctance [9,12] It has also been noted that erroneous information about vaccines was already being disseminated via the internet prior to the COVID-19 epidemic [13].

Consequently, we administered an online questionnaire to assess how pregnant women received knowledge about COVID-19 and immunization among vaccinated pregnant women. In this study, in addition to mass media, we also considered a variety of bidirectional communication media, such as internet media including websites and social media, and communication with their obstetricians.

## 2. Materials and Methods

### 2.1. Study Design and Participants

We conducted a cross-sectional study using an online national survey for pregnant women in Japan between 5 October and 22 November 2021, through the “Baby-plus” application (HEARZEST Co., Ltd., Yokohama, Japan), which is an application for pregnant women under the supervision of the Japan Society of Obstetrics and Gynecology (JSOG). Pregnant women can use Baby-Plus to keep a record of their pregnancy progress and obtain health information related to pregnancy. We invited pregnant women using Baby-Plus to participate in a questionnaire survey and surveyed those who voluntarily participated in the survey. Participants who were pregnant women older than 20 years, or married minors older than 16 years were recruited for the present study. Informed consent was obtained from all the potential participants prior to answering the questionnaire. The information was encrypted and converted into data through a secure server without identifying information. This study was approved by the Ethics Committee of Nihon University School of Medicine (approval number: 2021-04-02). All procedures were performed in accordance with the guidelines of our institutional ethics committee and adhered to the tenets of the Declaration of Helsinki. In the results of this survey, vaccination rates and side effects of the vaccine have already been published elsewhere [14]. The study required a minimum sample size of 384, based on a 95% confidence coefficient and a 5% margin of error, from a reference population of Japanese pregnant women.

### 2.2. Survey Questions

The online survey questionnaires included the characteristics and socioeconomic status of the pregnant women, such as age, weeks of gestation, primipara status, fetal number, pregnancy method, complications during pregnancy, and employment status. In addition, we asked about the methods they use to obtain information about COVID-19 and the COVID-19 vaccine.

Age was surveyed by selecting the age range of 16 to 45 years old, rather than having participants enter their age as a number. Analysis was conducted with the interpretation that the numerical value increases as age increases.

Regarding occupation, participants were asked to select one option from the following categories: “office worker”, “public servant”, “self-employed”, “educator”, “medical professional”, “housewife”, “part-time employee”, and “others”. The survey was conducted using a single-response format. For the variables used in the analysis, for example, “housewife” was set to 1, and all other options were set to 0. For “office worker”, this was set to 1, and the other options were set to 0. The same process was performed for other occupations. In the analysis, “housewife” was selected as the reference category.

The gestational weeks were determined by asking participants to select the corresponding numerical value for their current gestational week in response to the question, “Please tell us the gestational weeks you are today”. The question about the fetal number was “How many fetuses are you carrying in this pregnancy?” and offered the options of “Singleton” or “Multiple” to choose from. When asked “Is this your first pregnancy?”, the two choices given were “first pregnancy” or “second or more pregnancy”. For the method of conception, when asked “Which of the following applies to your current pregnancy?”, the options given were “natural conception”, “artificial insemination”, or “in vitro fertilization”.

For each media selection, participants were asked to choose one corresponding media from the following options in response to the question, “How did you research information about COVID-19 vaccine during pregnancy?”: “consulting their obstetrician-gynecologist”, “Japan Society of Obstetrics and Gynecology website, notifications from the Society”, “Japan Society of Obstetrics and Gynecology Infectious Diseases website, notifications from the Society”, “Japanese Ministry of Health, Labour and Welfare website”, “television/radio”, “newspapers”, “online news”, “YouTube”, “SNS of members of the National Diet”, “other SNS”, “information from family, friends, and acquaintances”, “did not specifically research”, “other”. Note that this item was a multiple-choice question. For the analysis, 11 media, excluding “did not specifically research” and “other” media, were targeted. For analysis, the websites issued by the Japan Society of Obstetrics and Gynecology, the Japan Society of Obstetrics and Gynecology Infectious Diseases, and the Japanese Ministry of Health, Labour, and Welfare were summarized as “professional medical website”, and television/radio, newspapers, and online news, which are a few reporting organizations disseminating information to an unspecified number of recipients, were summarized as “mass media”. YouTube, SNS of members of the National Diet, and other SNS were summarized as “social media”, and their sums were synthesized as composite variables for each category. Therefore, for these three summarized media, the value is 0 if none of them were used, and 3 if all of them were used, making it a four-level scale. Hence, a multiple regression analysis was conducted on these three summarized media. Additionally, the frequency of multimedia use, which uses multiple media simultaneously excluding the “did not specifically research” option, was also variable. This was a variable that synthesized the sum of 12 media, from consultations with regular obstetricians to other media.

The questions regarding anxiety about the risk of infection and vaccine risk were asked using the Likert scale for the following questions: “Please tell us the level of anxiety you have regarding COVID-19 infection” and “Please tell us the level of anxiety you have regarding the COVID-19 vaccine (mRNA vaccine)”. Respondents were asked to choose one of the following options: “Not anxious at all”, “Not very anxious”, “Neutral”, “Somewhat anxious”, or “Very anxious”.

For the question about the presence of disease under treatment, respondents were asked “Please tell us about any diseases you are currently being treated for”, and were given a list of options to choose from: “none”, “asthma”, “cancer”, “thyroid disease”, “autoimmune disease (SLE, rheumatoid arthritis, etc.)”, “allergies”, “inflammatory bowel disease (ulcerative colitis, Crohn’s disease, etc.)”, “hypertension”, “diabetes”, “heart disease”, “kidney disease”, “mental illness”, and “other”. Multiple answers were allowed for this question. To account for differences in media usage based on the presence or absence of illness, respondents who answered “none” were given a value of 1, while those who indicated any illness were given a value of 0.

Regarding pregnancy complications, we used a selection method where respondents chose from “none”, “COVID-19”, “gestational diabetes”, “preeclampsia”, “anemia”, “threatened miscarriage/preterm labor”, and “other” in response to the question “What illnesses did you contract during your current pregnancy?”. Multiple answers were allowed for this question. We assumed that the use of media would differ depending on the presence or absence of complications, so we assigned a value of 1 to “none” and 0 to respondents who had any complications.

### 2.3. Statistical Analyses

In this study, media selection was the dependent variable, and the independent variables included age, occupation, weeks of gestation, fetal number, first birth or otherwise, method of conception (natural or assisted reproduction), anxiety about COVID-19 infection risk, anxiety about the COVID-19 vaccine, presence of disease under treatment, and pregnancy complications. We conducted an exploratory binomial logistic regression analysis with each type of media use (including media non-use and frequency of multimedia use) as the dependent variable and multiple regression analysis with the number of vaccinations (three levels) as the dependent variable. Statistical analyses were performed using IBM SPSS Statistics 27 for Windows (IBM Japan, Tokyo, Japan); *p* values less than 0.05 were considered statistically significant.

## 3. Results

### 3.1. Characteristics of the Responders

**Figure 1:**
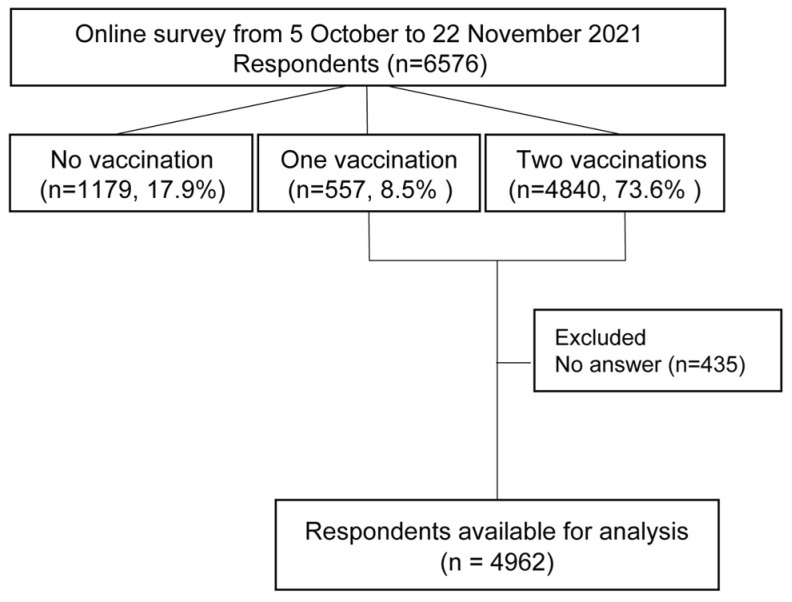
Flow diagram of the present study.

**Figure 2:**
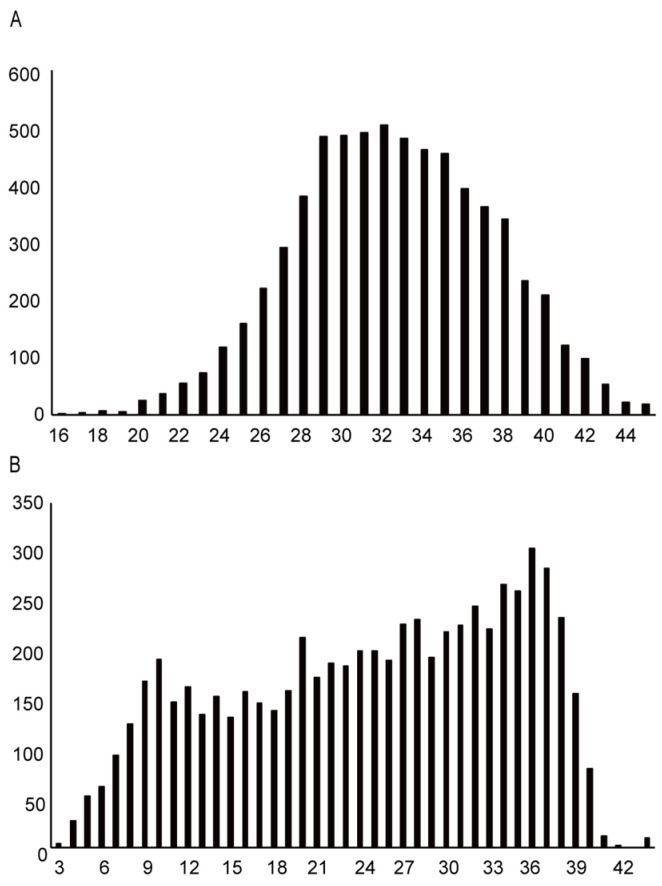
The distribution of age (**A**) and gestational weeks (**B**) of the participants.

**Figure 3:**
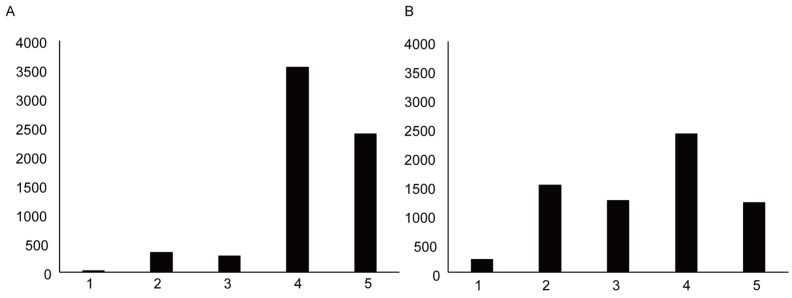
The distribution of the level of infection risk anxiety (**A**) and the level of vaccination risk anxiety (**B**) of the participants: (1) None, (2) Not very anxious, (3) Neutral, (4) Somewhat anxious, (5) Very anxious.

The flow diagram of the present study is shown in Figure 1. The number of participants in the study was 6576. Of the participants, 4840 (73.6%) were vaccinated twice, and 557 (8.5%) were vaccinated at least once. A total of 1179 (17.9%) responders had never been vaccinated against COVID-19. To analyse the media usage among pregnant women who received the vaccine, we removed 1179 individuals who had not received the vaccine. In addition, insufficient answers to the questionnaire were excluded (n = 435). The final number of responses available for analysis was 4962. The characteristics of the participant and descriptive statistics are presented in Table 1. The distribution of age (A) and gestational weeks (B) of the participants are shown in Figure 2. Figure 3 indicates the distribution of the level of infection risk anxiety (A) and vaccine risk anxiety (B) of the participants. Descriptive statistics are provided in Table S1. The total numbers of different media sites’ usage are shown in Table 2.

### 3.2. Characteristics of Pregnant Women Using Professional Medical Websites

Table 3 shows the results of a multiple regression analysis that includes characteristics of pregnant women who used the specialized medical websites of the Japanese Society of Obstetrics and Gynecology, the Japanese Society of Gynecology and Infectious Diseases, and the Ministry of Health, Labour, and Welfare (Japan). The results are presented as standardized regression coefficients (β), meaning that greater positive values indicated a greater likelihood that the participants were to use the media source, and greater negative values indicated that they were less likely to use the media source. In addition, since the standardized regression coefficients also allow for comparisons of effects with other variables, those with higher values had more influence on media selection than other independent variables.

Statistically significant characteristics of pregnant women who used professional medical websites were more frequently of older age (β 0.08, *p* < 0.001), in employment (β 0.05, *p* < 0.01) as public servants (β 0.07, *p* < 0.001) and medical professionals (β 0.03, *p* < 0.05), in later gestational weeks (β 0.10, *p* < 0.001), pregnancies through artificial insemination (β 0.05, *p* < 0.001) or in vitro fertilization (β 0.05, *p* < 0.001), and reporting stronger anxiety regarding infection risk (β 0.09, *p* < 0.001).

### 3.3. Characteristics of Pregnant Women Using Mass Media

Table 4 shows the results of the multiple regression analysis of pregnant women who used the three mass-media types, including TV/radio, newspapers, and online news.

The statistically significant characteristics of pregnant women who used mass media more were of older age (β 0.06, *p* < 0.001), in later gestational weeks (β 0.10, *p* < 0.001), with higher anxiety levels about infection risk (β 0.07, *p* < 0.001), and higher anxiety levels about vaccine risk (β 0.10, *p* < 0.001). Conversely, medical professionals were statistically significantly less likely to use mass media than the control group of housewives (β −0.09, *p* < 0.001).

### 3.4. Characteristics of Pregnant Women Using Social Media

Table 5 provides the results of the multiple regression analysis of the characteristics of pregnant women who used social media, such as YouTube, Twitter, Instagram, Facebook, the SNS accounts of the members of Congress, and other SNS accounts. The statistically significant characteristics of pregnant women who use social media more frequently were in later gestational weeks (β 0.06, *p* < 0.001), with higher anxiety levels about infection risk (β 0.07, *p* < 0.001), and higher anxiety levels about vaccine risk (β 0.05, *p* = 0.002). 

By occupation, compared to housewives, public servants (β −0.05, *p* < 0.01), educators (β −0.03, *p* < 0.05), and medical professionals (β −0.10, *p* < 0.001) were less likely to use social media to gather information on COVID-19 vaccines. 

### 3.5. Characteristics of Pregnant Women Using Multiple Types of Media

Table 6 shows the results of the analysis of the characteristics of pregnant women who used multiple media sources. Pregnant women, such as those who are older (β 0.07, *p* < 0.001), public servants (β 0.04, *p* < 0.05), in the late stage of pregnancy (β 0.20, *p* < 0.001), primiparous (β 0.04, *p* < 0.001), who have conceived through artificial insemination (β 0.06, *p* < 0.001) or in vitro fertilization (β 0.05, *p* < 0.001), who are highly concerned about the risks of the infection (β 0.121, *p* < 0.001) or vaccine (β 0.08, *p* < 0.001), tended to use multiple media sources to obtain information. On the other hand, medical professionals did not use multiple media sources (β −0.08, *p* < 0.001).

### 3.6. Characteristics of Pregnant Women Consulting Obstetricians

**Figure 4:**
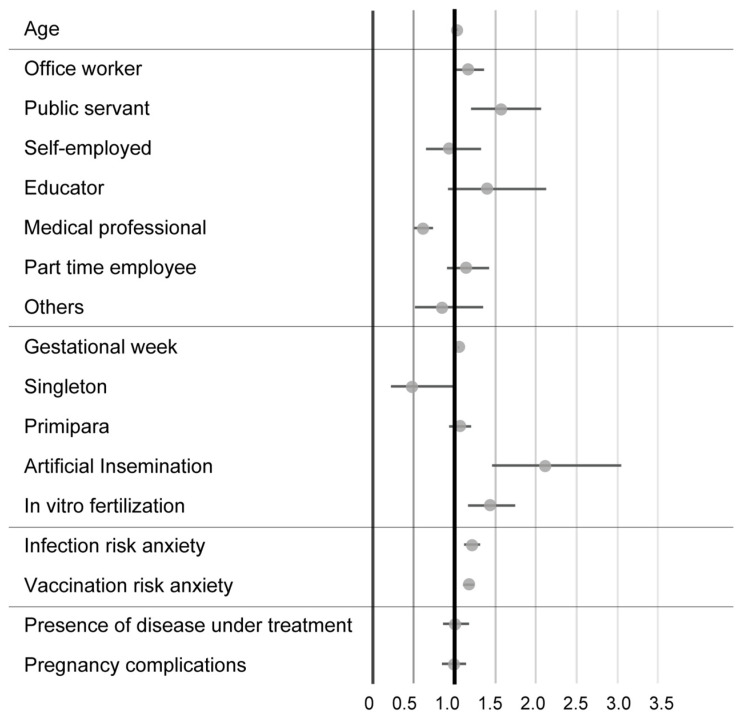
Characteristics of pregnant women who consult their obstetricians. The black circle indicates the odds ratio (OR), and the horizontal bar indicates the 95% confidence interval of the estimated value for each determinant.

Finally, we analysed the characteristics of pregnant women who consult with their obstetricians. The results are shown in Figure 4. The black circle for each item in the graph in Figure 2 indicates the odds ratio (OR), and the horizontal bar indicates the 95% confidence interval of the estimated value for each determinant. If the 95% confidence interval of an estimate straddles 1, it is not statistically significant. If the 95% confidence interval of the estimate lies above 1, it means a positive effect, and if it lies below 1, it means a negative effect.

The characteristics of pregnant women who answered that they often consult with their obstetrician were of older age (odds ratio 1.02, *p* < 0.01), public servants (odds ratio 1.57, *p* < 0.01), in the late stage of pregnancy (odds ratio 1.05, *p* < 0.001), or conceived through artificial insemination (odds ratio 2.11, *p* < 0.001) or in vitro fertilization (odds ratio 1.43, *p* < 0.001); in addition, infection risk anxiety (odds ratio 1.21, *p* < 0.001) and vaccine risk anxiety (odds ratio 1.17, *p* < 0.001) each had a positive effect on pregnant women consulting their obstetrician. On the other hand, medical professionals tended not to consult with their obstetrician (odds ratio 0.60, *p* < 0.001).

## 4. Discussion

The findings of this study indicate that pregnant women obtain information from a variety of media. In addition, there was a tendency for the media utilized to vary based on background. Although having proper knowledge about healthcare and medicine is important for a healthy life, the quality of the information, including its accuracy, depends on the type of media. This study revealed that pregnant women’s media consumption was influenced by variables such as their age, occupation, gestational week, mode of pregnancy, and fear of diseases or vaccinations. A correlation between health literacy and COVID-19 vaccination rates has been reported [15,16]. Health literacy refers to a person’s ability to receive, absorb, and comprehend the health information and services necessary to make educated decisions about their health. On the basis of the present survey’s findings, it is hypothesized that the level of health literacy of pregnant women is connected to their age and occupation, as these variables influence the techniques of information acquisition. There have already been many reports on SARS-CoV-2 infection during pregnancy and on COVID-19 vaccination [17,18,19,20,21,22]. It has also been shown that unvaccinated pregnant women were more susceptible to severe infections [10]. In addition, adverse reactions to the COVID-19 vaccine in pregnant women are comparable to those in nonpregnant women, and adverse reactions are not more severe because of pregnancy [14,22]. Moreover, COVID-19 vaccination in pregnant women did not have significant adverse effects on neonatal outcomes [22,23]. However, it is difficult for nonmedical experts or patients to obtain this important information easily because it has been reported in specialized scientific journals. A previous study by others revealed that pregnant women have hesitancy towards the COVID-19 vaccine, mainly caused by concerns related to adverse effects and misinformation on social media [24]. The other study reported that the advice of obstetricians was associated with high vaccination rates in pregnant women [25]. In addition, it was reported that pregnant women with higher education levels had higher vaccination rates [25]. It is suggested that women with higher education levels can obtain accurate information due to their higher level of media literacy. However, not only highly media-literate pregnant women but all pregnant women must be able to obtain accurate information appropriately. Hence, specialists are obligated to communicate information to pregnant women in an understandable manner. In fact, experts in maternal and child health, such as obstetricians and paediatricians, have distributed a great deal of information via websites and the media. In this study, it was found that many pregnant women collect information through professional medical websites and mass media. Particularly, older pregnant women, women who are approaching delivery, pregnant women who have become pregnant through assisted reproductive technology, and pregnant women with high levels of anxiety have been obtaining information from various media sources. These pregnant women may be assumed to have a high level of COVID-19 health literacy. However pregnant women who rely heavily on social media for information should be cautious. In recent years, with the development of the Internet and various applications, even people without specialized medical knowledge can easily share information on the Internet. Thus, inaccurate information based on incorrect knowledge is likewise mixed in with the Internet’s content [13,26]. Such erroneous information is thought to be detrimental to people’s health. During pandemics of infectious diseases, there is also the possibility of an infodemic [27].

An “infodemic” is a situation in which uncertain and inaccurate information spreads rapidly. According to the World Health Organization (WHO), the COVID-19-related infodemic can be as dangerous to human health and security as the pandemic itself [27,28]. An infodemic may cause confusion and inappropriate behaviour that can be harmful to health. It also leads to reduced trust in health authorities and medical professionals, which may prolong the pandemic. For example, during the COVID-19 pandemic, it was reported that anti-vaccination social media accounts were proliferating online [29]. Therefore, we must provide accurate information to nonexpert patients or pregnant women in an easy-to-understand manner. In addition, we should ensure that they are able to easily obtain the information. Since the beginning of the COVID-19 pandemic, the WHO, the CDC, and other institutions responsible for national health in each country, such as Japan’s Ministry of Health, Labour, and Welfare, and academic institutions such as universities and academic societies, have been making efforts to convey information about COVID-19 to nonspecialists in an easy-to-understand manner. However, since there have been some pregnant women who could not obtain appropriate information, it is also important to make efforts to publicize the ways in which they can obtain adequate information.

The usefulness of social media as an information tool for health and disease prevention has also been reported [30,31,32]. Public institutions and academic institutions, such as the WHO, CDC, National Institute of Health (NIH), universities, and the Ministry of Health, Labour, and Welfare have opened official social media accounts and are actively disseminating information. In addition, social media has the advantage of being able to have two-way communication. This advantage is being used to attempt various forms of support for pregnant women. In particular, the use of social media for mental health support for pregnant women has been reported to be effective [31,32]. For many people, social media has the advantage of being easy and casual to use, so it will be necessary to effectively utilize social media. In the present study, 21.8% of pregnant women were using social media to gather information about COVID-19 and COVID-19 vaccines. It is expected that more pregnant women will use social media to collect information about health and vaccines in the future. However, on the other hand, it has been reported that WHO is losing public credibility online for issues of extreme relevance to global health during the COVID-19 pandemic [33]. It is not a simple matter to convey information properly and appropriately to a large and diverse population. Further exploration will be needed on how to provide effective information.

This study has several limitations. First, it is a secondary analysis of data that has already been collected [14] and may not reflect changes in people’s understanding and acceptance of COVID-19 since the outbreak of the omicron strain, which is more infectious than the conventional strain. Second, this study was performed with pregnant Japanese women. The results of this survey are therefore restricted to the media consumption of pregnant women in Japan. As infodemics are a global issue, however, it may be necessary to research the methods of information searching used by pregnant women in countries other than Japan. For that, the findings of this study conducted in Japan would serve as a useful reference. Third, the study did not examine factors influencing willingness to be vaccinated, such as education and income. This study shows that older pregnant women are less reliant on social media sites and have a better understanding of vaccines, which may overlap with pregnant women with higher education levels and incomes. People with higher education levels are also more likely to have highly educated family and friends to consult. In contrast, women who give birth at a younger age may not have enough knowledgeable people available to them and may fall into an ‘echo chamber’ due to a lack of access to reliable information. Inaccurate information, such as anti-vaccine and coronavirus denial, is intentionally disseminated by certain countries in some cases; therefore, correct information must be provided to everyone for the safety of the population.

## 5. Conclusions

Experts in maternal and child health, such as obstetricians and paediatricians, should continue their efforts to provide pregnant women with understandable health information, including information about COVID-19 and vaccinations. In addition, it is essential that pregnant women must have easier access to this information.

## Figures and Tables

**Table 1:** Characteristics of the participants.

	Total (n = 6576)	
	n	%
COVID-19 vaccination		
None	1179	17.9
1	557	8.5
2	4840	73.6
Fetal number		
Single	6527	99.3
Twins or triplets	49	0.7
Primipara		
Yes	3780	57.5
No	2796	42.5
Pregnancy		
Natural	5540	84.2
Artificial insemination	224	3.4
In vitro fertilization	812	12.3
Complications during pregnancy		
Yes	1655	25.8
COVID-19	42	0.7
Gestational hypertension	47	0.7
Gestational diabetes mellitus	257	4.0
Anemia	786	12.3
Threatened premature delivery	596	9.3
Others	180	2.8
Diseases under treatment		
Yes	1071	17.6
Asthma	131	2.1
Malignancy	3	0.0
Thyroid disease	226	3.7
Autoimmune disease	33	0.5
Allergy	292	4.8
Inflammatory bowel disease	25	0.4
Hypertension	24	0.4
Diabetes mellitus	30	0.5
Heart disease	7	0.1
Kidney disease	10	0.2
Mental disorder	118	1.9
Others	300	4.9
Occupation		
Housewife	1514	23.0
Office worker	2470	37.6
Public servant	449	6.8
Self employed	218	3.3
Educator	148	2.3
Medical professional	889	13.5
Part-time employee	764	11.6
Others	124	1.9

**Table 2:** Websites used by participants to obtain information on COVID-19 and vaccines ^a^.

	Total (n = 5397)
Professional medical websites ^b^	6169
Mass media ^c^	4123
Social media ^d^	1178
Consulting obstetricians	3344
Others	1670
None	309

^a^ Multiple answers allowed. ^b^ The websites issued by the Japan Society of Obstetrics and Gynecology, the Japan Society of Obstetrics and Gynecology Infectious Diseases, and the Japanese Ministry of Health, Labour, and Welfare. ^c^ Television/radio, newspapers, and online news. ^d^ YouTube and SNS (Twitter, Instagram, Facebook, etc.).

**Table 3:** Characteristics of pregnant women using professional medical websites.

	Standardizing Coefficient (β)	Standard Error	*p* Value
Age	0.08	0.003	<0.001
Occupation			
Office worker	0.05	0.04	0.004
Public servant	0.07	0.06	<0.001
Self-employed	0.03	0.09	0.069
Educator	0.03	0.10	0.077
Medical professional	0.03	0.05	0.041
Part-time employee	−0.02	0.05	0.169
Others	0.01	0.12	0.321
Gestational week	0.10	0.002	<0.001
Singleton	0.01	0.16	0.68
Primipara	0.02	0.03	0.263
Artificial insemination	0.05	0.08	<0.001
In vitro fertilization	0.05	0.05	<0.001
Infection risk anxiety	0.09	0.02	<0.001
Vaccination risk anxiety	−0.03	0.01	0.094
Presence of disease under treatment	−0.02	0.04	0.168
Pregnancy complications	0.01	0.04	0.484

**Table 4:** Characteristics of pregnant who women use mass media.

	Standardizing Coefficient (β)	Standard Error	*p* Value
Age	0.06	0.003	<0.001
Occupation			
Office worker	−0.011	0.03	0.541
Public servant	0.01	0.05	0.694
Self-employed	0.002	0.07	0.874
Educator	0.021	0.08	0.152
Medical professional	−0.09	0.04	<0.001
Part-time employee	−0.02	0.04	0.329
Others	0.01	0.10	0.62
Gestational week	0.10	0.001	<0.001
Singleton	−0.01	0.13	0.417
Primipara	0.02	0.03	0.189
Artificial insemination	0.02	0.06	0.135
In vitro fertilization	0.02	0.04	0.326
Infection risk anxiety	0.07	0.02	<0.001
Vaccination risk anxiety	0.10	0.01	<0.001
Presence of disease under treatment	0.01	0.03	0.326
Pregnancy complications	0.01	0.03	0.732

**Table 5:** Characteristics of pregnant who women use social media.

	Standardizing Coefficient (β)	Standard Error	*p* Value
Age	−0.03	0.001	0.059
Occupation			
Office worker	−0.02	0.02	0.227
Public servant	−0.05	0.03	0.003
Self-employed	0.03	0.04	0.074
Educator	−0.03	0.04	0.046
Medical professional	−0.10	0.02	<0.001
Part-time employee	−0.02	0.02	0.305
Others	0.02	0.05	0.152
Gestational week	0.06	0.001	<0.001
Singleton	−0.01	0.07	0.659
Primipara	0.02	0.01	0.112
Artificial insemination	0.03	0.04	0.051
In vitro fertilization	0.01	0.02	0.38
Infection risk anxiety	0.07	0.01	<0.001
Vaccination risk anxiety	0.05	0.01	0.002
Presence of disease under treatment	0.001	0.02	0.936
Pregnancy complications	0.03	0.02	0.020

**Table 6:** Characteristics of pregnant women who used multiple media sources.

	Standardizing Coefficient (β)	Standard Error	*p* Value
Age	0.07	0.006	<0.001
Occupation			
Office worker	0.02	0.068	0.331
Public servant	0.038	0.109	0.011
Self-employed	0.027	0.153	0.060
Educator	0.024	0.170	0.085
Medical professional	−0.080	0.086	<0.001
Part-time employee	−0.025	0.093	0.111
Others	0.014	0.205	0.324
Gestational week	0.198	0.003	<0.001
Singleton	−0.010	0.282	0.462
Primipara	0.036	0.054	0.013
Artificial insemination	0.060	0.137	<0.001
In vitro fertilization	0.053	0.082	<0.001
Infection risk anxiety	0.121	0.035	<0.001
Vaccination risk anxiety	0.082	0.025	<0.001
Presence of disease under treatment	−0.001	0.067	0.947
Pregnancy complications	0.018	0.061	0.216

## Data Availability

The authors declare that the data supporting the findings of this study are available within the paper.

## Supplementary Materials

The following are available online at https://www.mdpi.com/article/10.3390/vaccines11040805/s1, Table S1: Descriptive statistics.
