# Supplementary material for: The Factors Influencing Pregnant Women’s Selection of Media Sources to Obtain Information on COVID-19 in Japan in 2021
Source: Vaccines (Basel). 2023 Apr 6;11(4):805. doi: 10.3390/vaccines11040805 (PMC10143418; doi:10.3390/vaccines11040805)
Supplement: Supplementary file 1 [file vaccines-11-00805-s001.zip › vaccines-2250814-supplementary.pdf]

Table S1. Descriptive statistics.

	Frequency	Minimum	Maximum	Mean	SD
Consulting obstetricians	5397	0.00	1.00	0.6196	0.48553
Medical specialized web media	5397	1.00	4.00	2.1430	1.03422
Mass media	5397	1.00	4.00	1.7639	0.84789
Social media	5397	1.00	4.00	1.2183	0.45617
Multimedia	5397	0.00	10.00	3.0543	1.85896
Age	6576	1	30	17.49	4.861
Office worker	6576	0.00	1.00	0.3756	0.48432
Public servant	6576	0.00	1.00	0.0683	0.25224
Self-employed	6576	0.00	1.00	0.0332	0.17904
Educator	6576	0.00	1.00	0.0225	0.14833
Medical professional	6576	0.00	1.00	0.1352	0.34195
Part-time employee	6576	0.00	1.00	0.1162	0.32047
Others	6576	0.00	1.00	0.0189	0.13603
Gestational week	6576	1	42	22.71	9.783
Singleton	6576	0.00	1.00	0.9925	0.08601
Primipara	6576	0.00	1.00	0.5748	0.49441
Artificial insemination	6576	0.00	1.00	0.0341	0.18141
In vitro fertilization	6576	0.00	1.00	0.1235	0.32901
Infection risk anxiety	6576	1	5	4.20	0.780
Vaccination risk anxiety	6576	1	5	3.43	1.130
Presence of disease under treatment	6094	0.00	1.00	0.8243	0.38064
Pregnancy complications	6416	0.00	1.00	0.7421	0.43754
Number of Effective Cases	4962				

SD, Standard Deviation
